# Psychosocial pathways linking type D personality traits to quality of life in patients with permanent intestinal stomas: a cross-sectional structural equation modeling study

**DOI:** 10.3389/fmed.2026.1835262

**Published:** 2026-06-03

**Authors:** Chen Tan, Hongyu Zhang, Yueyang Jiang, Yanyan Zhang, Meiqi Yu

**Affiliations:** 1 Department of Endocrinology and Metabolism, The First Hospital of China Medical University, Shenyang, China; 2 Department of Hepatic-biliary Surgery, The First Hospital of China Medical University, Shenyang, China; 3 Department of Cardiac Surgery, The First Hospital of China Medical University, Shenyang, Liaoning, China

**Keywords:** permanent intestinal stoma, quality of life, self-care ability, self-efficacy, social support, structural equation modeling, type D personality traits

## Abstract

**Background:**

Patients with permanent intestinal stomas often experience long-term physical and psychosocial challenges, including lifelong appliance management, altered body image, social embarrassment, and self-care burden, which can adversely affect their quality of life. Because Type D personality traits are characterized by negative affectivity and social inhibition, they may be particularly relevant to psychosocial adaptation in this population. However, the potential psychosocial and behavioral pathways linking Type D personality traits to quality of life in patients with permanent intestinal stomas remain unclear.

**Methods:**

This cross-sectional study included 200 patients with permanent intestinal stomas recruited from a tertiary hospital. All major variables were assessed using self-report questionnaires, including the 14-item Type D Scale (DS14), the Social Support Rating Scale (SSRS), the General Self-Efficacy Scale (GSES), the Ostomy Self-Care Index (OSCI), and the Stoma Quality of Life Scale (Stoma-QOL). Pearson correlation analysis, structural equation modeling, and bootstrap analysis with 5,000 resamples were performed to examine associations and indirect effects.

**Results:**

Higher Type D personality trait scores were negatively associated with social support, self-efficacy, stoma self-care ability, and quality of life. The structural equation model showed an acceptable fit to the data (χ^2^/df = 2.63, CFI = 0.92, TLI = 0.90, RMSEA = 0.071, SRMR = 0.061). Self-efficacy significantly mediated the association between Type D personality traits and quality of life. The sequential pathway involving social support and self-efficacy was also statistically significant. In contrast, the indirect pathway through social support alone and the longer sequential pathway through social support, self-efficacy, and stoma self-care ability did not reach statistical significance.

**Conclusion:**

Stronger Type D personality traits were associated with lower quality of life in patients with permanent intestinal stomas. These findings suggest partial statistical mediation within a cross-sectional SEM framework, particularly through self-efficacy, rather than confirmed causal mechanisms. Self-efficacy and supportive resources may be useful targets for future longitudinal and intervention studies aimed at improving quality of life in this population.

## 1 Introduction

Colorectal cancer and other severe gastrointestinal diseases often require surgical interventions that result in the creation of a permanent intestinal stoma (1–3). Although stoma surgery can be life-saving, a permanent stoma alters the natural pathway of fecal excretion and requires lifelong appliance management. Patients must adapt to daily stoma care, dietary adjustment, altered body image, and concerns about leakage, odor, social interaction, and intimacy (4–9). These long-term demands may reduce quality of life and contribute to psychological distress, including anxiety and depression (7, 8, 10, 11).

Because adaptation to a permanent stoma involves both psychosocial adjustment and self-management, quality of life in this population may be shaped by the interaction of personality-related emotional tendencies, interpersonal resources, self-efficacy, and practical self-care ability (12–19). These features make patients with permanent intestinal stomas a clinically relevant population in which to examine how Type D personality traits may be statistically associated with social support, self-efficacy, stoma self-care ability, and quality of life within a cross-sectional pathway model.

Among psychological characteristics, Type D personality traits have attracted growing interest in health psychology research. Type D personality, first proposed by Denollet, is characterized by two stable traits: negative affectivity and social inhibition (20). Individuals with high negative affectivity tend to experience persistent negative emotions such as worry, irritability, and pessimism. At the same time, social inhibition reflects a tendency to avoid social interactions due to fear of rejection or disapproval. These characteristics may limit individuals’ ability to seek support from others and to cope effectively with stressful life events (21, 22).

A growing body of research has demonstrated that Type D personality traits are associated with adverse health outcomes across a range of chronic diseases and medical populations (21, 22). Patients with Type D personality traits are more likely to report higher levels of emotional distress, poorer psychological adjustment, and lower quality of life (13, 21, 22). Despite the growing literature on Type D personality traits in medical populations, relatively few studies have examined its impact on patients living with permanent intestinal stomas.

Nevertheless, Type D personality traits may overlap with broader psychological distress, particularly because the negative affectivity component is conceptually related to depressive symptoms, anxiety, and general distress. In this cross-sectional study, we therefore considered Type D personality traits as risk-related psychosocial characteristics rather than definitive causal personality determinants. This cautious interpretation is especially relevant in stoma populations, in whom postoperative distress, body image concerns, and social embarrassment may influence negative affectivity and social inhibition.

Social support refers to the emotional, informational, and practical assistance that individuals receive from family members, friends, healthcare providers, and the broader community (12, 23).

For individuals with permanent intestinal stomas, social support may play a particularly important role. Adjusting to life with a stoma often requires learning new self-care skills, managing potential complications, and overcoming concerns related to body image and social acceptance (4–10). Support from family members and healthcare professionals may provide reassurance, guidance, and practical assistance during this adaptation process. Previous studies have shown that patients with stronger social support networks tend to report better psychological adjustment and improved quality of life (4–8, 11, 12, 23).

Another key factor influencing patients’ health behaviors is self-efficacy, a concept derived from social cognitive theory. Self-efficacy refers to individuals’ beliefs in their ability to successfully perform specific behaviors and manage challenging situations (24).

For patients with permanent intestinal stomas, self-efficacy is closely related to their ability to perform daily stoma care tasks. Effective stoma management involves a range of practical skills, including cleaning the stoma, changing appliances, protecting the peristomal skin, and monitoring for complications (14–18, 25). Patients who possess higher levels of self-efficacy are more likely to actively engage in these self-care activities and to respond effectively when problems arise (13–18, 25). Consequently, self-efficacy may serve as an important psychological potential pathway linking psychosocial resources to quality of life (13, 15).

Closely related to self-efficacy is stoma self-care ability, which represents patients’ actual capacity to perform daily stoma management behaviors. Adequate self-care ability can reduce the risk of complications, improve physical comfort, and increase patients’ independence in daily life (14, 16–18, 25).

Conceptually, social support, self-efficacy, and stoma self-care ability may represent different but connected levels of adaptation. Social support can be regarded as an external interpersonal resource, self-efficacy as an internal psychological resource, and stoma self-care ability as the behavioral enactment of confidence in daily stoma management. The proposed ordering was theoretically informed rather than empirically proven as a temporal sequence. Type D personality traits were placed upstream because they reflect emotional and interpersonal tendencies that may affect patients’ willingness to seek or use support. Under this framework, stronger Type D personality traits may be statistically associated with lower perceived or utilized support, weaker self-efficacy, poorer self-care ability, and lower quality of life. Given the cross-sectional design, this ordering should be interpreted as a theoretical statistical pathway rather than a confirmed causal sequence.

Although previous studies have independently examined the roles of personality traits, social support, and self-efficacy in health outcomes, relatively few studies have integrated these variables within a single analytical framework. In particular, the potential psychological and behavioral pathways linking Type D personality traits to quality of life in patients with permanent intestinal stomas remain insufficiently understood (13–17, 21, 22). To address this gap, the present study developed a structural equation model to explore the relationships among Type D personality traits, social support, self-efficacy, stoma self-care ability, and quality of life. Specifically, we hypothesized that Type D personality traits would be negatively associated with quality of life and that this relationship would be mediated by psychosocial and behavioral factors.

The specific hypotheses of the study were as follows: Type D personality traits are negatively associated with quality of life in patients with permanent intestinal stomas. Social support is positively associated with self-efficacy and quality of life. Self-efficacy would be positively associated with stoma self-care ability and quality of life. Social support, self-efficacy, and stoma self-care ability would statistically mediate the relationship between Type D personality traits and quality of life. By examining these relationships within a comprehensive structural model, this study aims to provide a deeper understanding of the potential psychosocial and behavioral pathways associated with quality of life in patients with permanent intestinal stomas. The findings may also provide valuable evidence for informing future longitudinal or intervention research to improve psychosocial adaptation and long-term health outcomes in this patient population. Given the cross-sectional design, these hypotheses were tested as theoretical statistical associations rather than as evidence of confirmed temporal or causal relationships.

## 2 Materials and methods

### 2.1 Study design and participants

This cross-sectional study was conducted to examine the relationships among Type D personality traits, social support, self-efficacy, stoma self-care ability, and quality of life in patients with permanent intestinal stomas. Participants were recruited from the First Hospital of China Medical University between January 2023 and December 2025. The study population profile and care context are generally consistent with recent stoma-related observational and quality-of-life studies (7, 8, 10, 11).

Inclusion criteria: (1) aged 18 years or older; (2) had undergone permanent intestinal stoma surgery due to colorectal disease; (3) had a postoperative stoma duration of at least 3 months; (4) were able to understand and complete the questionnaire independently or with assistance.

Patients were excluded if they had severe cognitive impairment, diagnosed psychiatric disorders, or serious medical complications that could interfere with questionnaire completion.

A total of 220 patients were initially approached, among whom 200 agreed to participate and completed the questionnaires, yielding a response rate of 90.9%. Given that the hypothesized model used five observed composite variables and a limited number of structural paths, the final sample size of 200 was considered adequate for the observed-variable path analysis and bootstrap mediation testing.

### 2.2 Ethical considerations

This study was approved by Ethics Committee of the First Hospital of China Medical University. All procedures were conducted in accordance with the ethical principles outlined in the World Medical Association Declaration of Helsinki. Written informed consent was obtained from all participants prior to data collection. Participants were informed that their responses would remain confidential and would only be used for research purposes.

### 2.3 Measures

#### 2.3.1 Type D personality traits

Type D personality traits were assessed using the 14-item Type D Scale (DS14), developed by Denollet (20). The DS14 comprises two 7-item subscales: Negative Affectivity (NA) and Social Inhibition (SI). Each item is rated on a 5-point Likert scale from 0 (“false”) to 4 (“true”), yielding subscale scores ranging from 0 to 28. Higher scores indicate stronger negative affectivity and social inhibition. According to the conventional categorical scoring approach, participants with scores of ≥ 10 on both the NA and SI subscales can be classified as having Type D personality.

In the present study, the conventional cutoff was used to describe the proportion of participants meeting the criteria for Type D personality classification, while the DS14 total score was used as a continuous indicator of Type D personality traits in the primary correlation and structural equation modeling analyses. This continuous operationalization was chosen because the primary objective of this study was to examine graded psychosocial and behavioral pathways rather than to compare categorical Type D and non-Type D groups. In addition, treating DS14 scores continuously allowed more information to be retained and avoided the potential loss of statistical power associated with dichotomizing continuous trait scores.

Based on the conventional DS14 cutoff of ≥ 10 on both the NA and SI subscales, 68 participants (34.0%) were classified as having Type D personality. In the present study, Cronbach’s α coefficients were 0.84 for the total scale, 0.82 for the NA subscale, and 0.79 for the SI subscale.

#### 2.3.2 Social support

Perceived social support, which has consistently been associated with better psychological adjustment, stronger self-efficacy, and improved quality of life (10–12, 23) was measured using the Social Support Rating Scale (SSRS), developed by Xiao Shuiyuan for use in Chinese populations (26). The SSRS contains 10 items across three dimensions: subjective support, objective support, and support utilization. The total score ranges from 12 to 66, with higher scores indicating greater perceived social support. The SSRS has been widely used in Chinese psychosocial and medical research. In the present study, the Cronbach’s α coefficient for the total scale was 0.76.

#### 2.3.3 Self-efficacy

Self-efficacy was assessed using the General Self-Efficacy Scale (GSES) developed by Schwarzer and Jerusalem (24). The GSES consists of 10 items rated on a 4-point Likert scale from 1 (“not at all true”) to 4 (“exactly true”). Total scores range from 10 to 40, with higher scores indicating stronger generalized self-efficacy, which associated with better self-management behaviors and improved health-related quality of life (13, 15). The scale has shown satisfactory reliability across different populations and languages. In the present study, the Cronbach’s α coefficient for the GSES was 0.88. The GSES was used to represent generalized self-efficacy in the primary model because this study focused on self-efficacy as a broader psychosocial resource rather than only stoma-specific confidence in self-care tasks.

#### 2.3.4 Stoma self-care ability

Stoma self-care ability was measured using the Ostomy Self-Care Index (OSCI), a 32-item instrument developed to assess self-care in patients with ostomy. The OSCI includes four domains: self-care maintenance, self-care monitoring, self-care management, and self-care self-efficacy. Items are rated on a 5-point Likert scale, with higher scores indicating better self-care ability. Current research indicates that intervention and self-management studies have shown that stronger self-care ability is associated with improved stoma adaptation, fewer complications, and better quality of life (14, 16–18, 25). Because generalized self-efficacy was separately assessed using the GSES, the primary SEM model used only the behavioral domains of the OSCI, namely self-care maintenance, self-care monitoring, and self-care management, to represent stoma self-care ability. The OSCI self-care self-efficacy domain was not included in the primary self-care ability construct to maintain clearer conceptual separation between generalized self-efficacy and behavioral stoma self-care ability. However, because generalized self-efficacy and stoma-specific self-care self-efficacy may represent related but distinct constructs, we further examined their correlation and conducted a sensitivity analysis. In the present study, the Cronbach’s α coefficient for the self-care domains used in the analysis was 0.81.

#### 2.3.5 Quality of life

Quality of life was measured using the Stoma Quality of Life Scale (Stoma-QOL), originally developed and validated by Prieto and colleagues for patients with colostomy or ileostomy (27). Studies in ostomy populations have shown that quality of life is closely related to adaptation, symptom burden, psychosocial adjustment, and self-management capacity (4–8, 28). The Stoma-QOL is a stoma-specific instrument consisting of 20 items derived from an initial larger item pool during instrument development. Items are rated on a 4-point response format, and higher transformed scores indicate better stoma-related quality of life. The scale has been used internationally and has shown acceptable validity and reliability in ostomy populations. In the present study, the Cronbach’s α coefficient for the total scale was 0.90.

### 2.4 Data collection

Data were collected using structured self-administered questionnaires. Trained nurses explained the study objectives and provided instructions for completing the questionnaires. Participants completed the questionnaires in a quiet environment within the clinic. For patients with visual or literacy difficulties, trained staff provided assistance by reading the questions aloud and recording responses. Similar data collection procedures have been used in recent studies on stoma-related psychosocial outcomes (7, 8).

### 2.5 Statistical analysis

All statistical analyses were performed using SPSS 26.0 and AMOS 24.0. Descriptive statistics were used to summarize demographic and clinical characteristics. Continuous variables were expressed as mean ± standard deviation (SD), and categorical variables were presented as frequencies and percentages. Before the main analyses, the dataset was checked for completeness, distribution characteristics, and outliers. Cases with substantial missing information were excluded during questionnaire screening, and the final dataset used for analysis contained complete data for all study variables. The distributions of continuous variables were examined using means, standard deviations, observed ranges, skewness, and kurtosis, and no serious deviations were identified that would preclude maximum-likelihood estimation in AMOS. Pearson correlation analysis was conducted to examine the relationships among Type D personality traits, social support, self-efficacy, stoma self-care ability, and quality of life. The DS14 total score was treated as a continuous variable in the main correlation and SEM analyses. The conventional categorical classification of Type D personality was used only for descriptive purposes. To address the potential distinction between generalized self-efficacy and stoma-specific self-care self-efficacy, we additionally examined the Pearson correlation between the GSES score and the OSCI self-care self-efficacy domain. A sensitivity analysis was further conducted by replacing the GSES score with the OSCI self-care self-efficacy domain in the structural equation model, while retaining the behavioral OSCI domains as the measure of stoma self-care ability.

To reduce the potential impact of conceptual overlap in the structural model, generalized self-efficacy was represented by the GSES, whereas stoma self-care ability was represented by the behavioral domains of the OSCI only; the OSCI self-care self-efficacy domain was not included in the structural equation modeling (SEM) analysis. The SEM was conducted as an observed-variable path analysis using composite scale scores rather than as a full latent-variable SEM with item-level measurement models. SEM was used to test the hypothesized mediation model. Model fit was evaluated using the following indices: χ^2^/df, CFI, TLI, RMSEA, and SRMR. Acceptable model fit was defined as CFI ≥ 0.90, TLI ≥ 0.90, RMSEA ≤ 0.08, and SRMR ≤ 0.08. Direct, indirect, and total effects were estimated within the same path model. Indirect effects were evaluated using bootstrap analysis with 5000 resamples and 95% confidence intervals. No formal comparison with alternative directional models was performed; therefore, the proposed model was interpreted as a theoretically informed cross-sectional path model rather than as empirically confirmed evidence of temporal or causal ordering. In addition, collinearity among the major study variables was reviewed before model estimation, and no evidence of problematic overlap was observed at the correlation level. Because all major variables were collected using self-reported questionnaires, procedural and statistical approaches were used to reduce and assess common method bias. During data collection, anonymity, confidentiality, and standardized instructions were emphasized. Harman’s single-factor test was conducted as a supplementary assessment of common method bias. Specifically, the number of factors with eigenvalues greater than 1.0 and the percentage of variance explained by the first unrotated factor were examined. In addition, a common latent factor approach was performed to further assess whether common method variance substantially influenced the main SEM results. This analytic strategy is consistent with recent SEM-based and self-management-focused studies in ostomy and chronic disease populations (14–17).

## 3 Results

### 3.1 Participant characteristics

A total of 200 patients with permanent intestinal stomas were included in this study. The demographic and clinical characteristics of the participants are presented in Table 1. The mean age of the participants was 56.3 ± 12.4 years, and the majority of participants were male. Most patients had undergone stoma surgery due to colorectal cancer, and a large proportion had lived with the stoma for more than 1 year. Overall, these characteristics suggest that the study sample is broadly representative of patients with permanent intestinal stomas receiving follow-up care in tertiary hospital.

**TABLE 1:** Demographic and clinical characteristics of the participants (*n* = 200).

Variable	Category	*n* (%)
Gender	Male	118 (59.0)
	Female	82 (41.0)
Age	< 50 years	46(23.0)
	50–65 years	96 (48.0)
	> 65 years	58(29.0)
Marital status	Married	156 (78.0)
	Other	44 (22.0)
Education level	Primary school or below	52 (26.0)
	Secondary school	84 (42.0)
	College or above	64 (32.0)
Stoma duration	3–12 months	72 (36.0)
	> 12 months	128 (64.0)

Harman’s single-factor test was conducted to provide a supplementary assessment of common method bias. The analysis identified seven factors with eigenvalues greater than 1.0. The first unrotated factor explained 31.0% of the total variance, which did not account for the majority of the variance. In addition, the common latent factor analysis showed that adding a common latent factor did not materially change the direction or interpretation of the main standardized path coefficients, with changes in the main path estimates all below 0.18. These findings suggested that common method variance was unlikely to substantially bias the main results.

### 3.2 Descriptive statistics of the main study variables

The descriptive statistics of the main study variables are presented in Table 2. The mean DS14 total score was 18.7 ± 8.1, indicating a moderate level of Type D personality traits in the study sample. Based on the conventional DS14 cutoff of ≥ 10 on both the NA and SI subscales, 68 participants (34.0%) were classified as having Type D personality. The mean score of social support was 39.2 ± 8.6, suggesting a moderate level of perceived support among the participants. The mean score of self-efficacy was 24.8 ± 5.7, while the mean score of stoma self-care ability was 66.1 ± 12.9. The average quality of life score was 58.4 ± 14.7, reflecting a moderate level of stoma-related quality of life among patients with permanent intestinal stomas.

**TABLE 2:** Descriptive statistics of the main study variables.

Variable	Mean	SD	Min	Max
Type D personality traits (DS14 total score)	18.7	8.1	3	41
Social support (SSRS)	39.2	8.6	18	61
Self-efficacy (GSES)	24.8	5.7	11	39
Stoma self-care ability	66.1	12.9	31	93
Quality of life (Stoma-QOL transformed)	58.4	14.7	22	89

DS14, 14-item Type D Scale. The DS14 total score was used as a continuous indicator of Type D personality traits in the primary analyses. Based on the conventional cutoff of ≥ 10 on both the NA and SI subscales, 68 participants (34.0%) were classified as having Type D personality.

### 3.3 Correlation analysis

Pearson correlation analysis was performed to examine the relationships among Type D personality traits, social support, self-efficacy, stoma self-care ability, and quality of life. The results are presented in Table 3. Higher Type D personality trait scores were significantly negatively correlated with social support, self-efficacy, stoma self-care ability, and quality of life (all *p* < 0.01). In contrast, social support was positively correlated with self-efficacy, stoma self-care ability, and quality of life. Self-efficacy was also positively associated with stoma self-care ability and quality of life. These findings indicate that psychosocial resources and behavioral capabilities are closely related to quality of life among patients with permanent intestinal stomas and support the subsequent mediation analysis.

**TABLE 3:** Pearson correlation matrix for the main study variables.

Variable	1	2	3	4	5
1 Type D personality traits	1	1	1	1	1
2 Social support	−0.29**				
3 Self-efficacy	−0.33**	0.36**			
4 Stoma self-care ability	−0.18*	0.21**	0.43**		
5 Quality of life	−0.38**	0.34**	0.46**	0.31**	

*p < 0.05;

**p < 0.01.

### 3.4 Correlation and sensitivity analysis of self-efficacy measures

The GSES score was moderately correlated with the OSCI self-care self-efficacy domain (*r* = 0.47, *p* < 0.001), indicating that generalized self-efficacy and stoma-specific self-care self-efficacy were related but not identical constructs. In the sensitivity analysis, replacing the GSES score with the OSCI self-care self-efficacy domain did not materially change the direction or interpretation of the main pathways. These findings supported the use of the GSES score as the primary indicator of generalized self-efficacy in the main structural equation model.

### 3.5 Structural equation modeling

To further examine the relationships among the study variables, structural equation modeling was conducted. The hypothesized model included Type D personality traits, represented by the DS14 total score, as the independent variable, quality of life as the dependent variable, and social support, self-efficacy, and stoma self-care ability as mediating variables. The model fit indices are shown in Table 4. The results indicated that the structural equation model demonstrated an acceptable fit to the data (χ^2^ = 28.93, df = 11, *p* = 0.002, χ^2^/df = 2.63, CFI = 0.92, TLI = 0.90, RMSEA = 0.071, and SRMR = 0.061), suggesting that the proposed model adequately represented the observed relationships among the variables.

**TABLE 4:** Fit indices for the structural equation model.

Fit index	Value	Recommended value
χ^2^	28.93	–
df	11	–
*p*-value for χ^2^ test	0.002	–
χ^2^/df	2.63	< 3
CFI	0.92	≥ 0.90
TLI	0.90	≥ 0.90
RMSEA	0.071	≤ 0.08
SRMR	0.061	≤ 0.08

**FIGURE 1:**
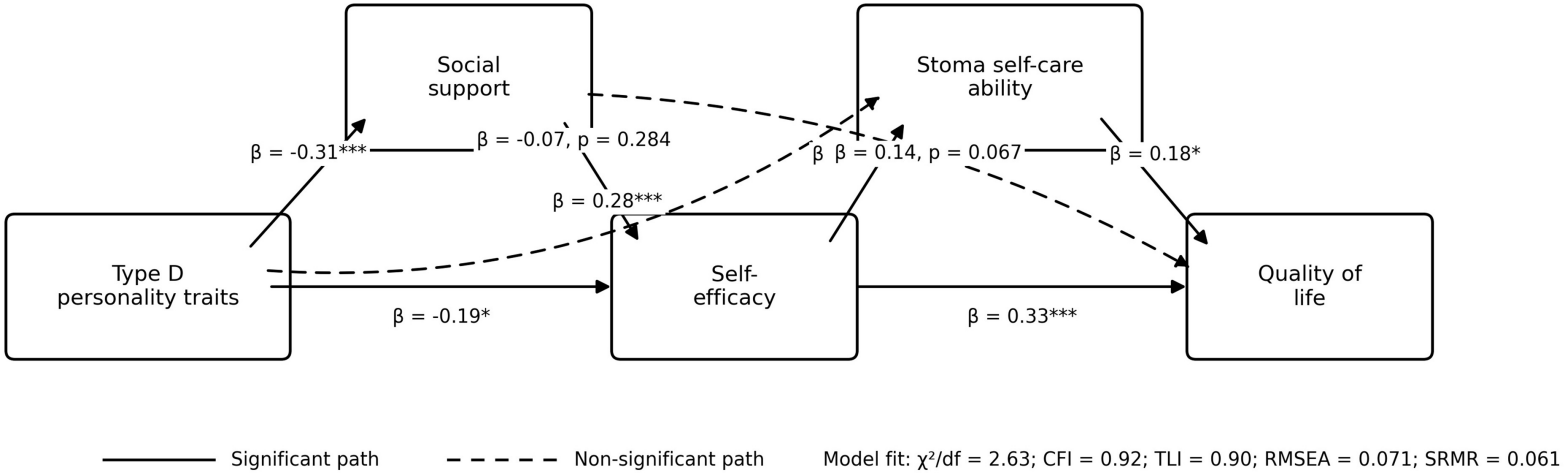
Structural equation model of the psychosocial and self-care pathways linking Type D personality traits to quality of life in patients with permanent intestinal stomas. Standardized path coefficients are shown. Solid lines indicate statistically significant paths, and dashed lines indicate non-significant paths. Model fit: χ^2^/df = 2.63, CFI = 0.92, TLI = 0.90, RMSEA = 0.071, and SRMR = 0.061. *p < 0.05; ***p < 0.001.

As shown in Figure 1, higher Type D personality trait scores were negatively associated with social support (β = –0.31, *p* < 0.001) and self-efficacy (β = –0.19, *p* = 0.012), but were not significantly associated with stoma self-care ability (β = –0.07, *p* = 0.284). Social support was positively associated with self-efficacy (β = 0.28, *p* < 0.001), while its direct association with quality of life was not statistically significant (β = 0.14, *p* = 0.067). Self-efficacy was positively associated with both stoma self-care ability (β = 0.39, *p* < 0.001) and quality of life (β = 0.33, *p* < 0.001). Stoma self-care ability was also positively associated with quality of life (β = 0.18, *p* = 0.021). The non-significant direct path from Type D personality traits to stoma self-care ability suggests that Type D personality traits may not be directly linked to behavioral self-care capacity once social support and self-efficacy are included in the model.

### 3.6 Mediation analysis

To further examine the mediating effects of social support, self-efficacy, and stoma self-care ability, bootstrap analysis with 5,000 resamples was conducted. The results are presented in Table 5. Type D personality trait scores had significant indirect effects on quality of life through multiple pathways. Specifically, the indirect pathway through self-efficacy and the sequential pathway through social support and self-efficacy were statistically significant. A chain mediation pathway was observed from Type D personality traits through social support, self-efficacy, and stoma self-care ability to quality of life.

**TABLE 5:** Bootstrap analysis of indirect effects.

Pathway	Standardized indirect effect	95% CI	Interpretation
Type D personality traits → Social support → QOL	−0.04	−0.10 to 0.01	Not statistically significant
Type D personality traits → Self-efficacy → QOL	−0.06	−0.12 to −0.02	Statistically significant
Type D personality traits → Social support → Self-efficacy → QOL	−0.03	−0.07 to −0.01	Statistically significant
Type D personality traits → Self-efficacy → Self-care → QOL	−0.01	−0.04 to −0.001	Statistically significant
Type D personality traits → Social support → Self-efficacy → Self-care → QOL	−0.01	−0.03 to 0.00	Not statistically significant

CI, confidence interval; QOL, quality of life. Indirect effects were tested using bootstrap analysis with 5,000 resamples. An indirect effect was considered statistically significant when the 95% CI did not include zero.

As shown in Table 5, the indirect effect of Type D personality trait scores on quality of life through self-efficacy was statistically significant. The sequential indirect pathway from Type D personality trait scores to quality of life through social support and self-efficacy was also significant. In addition, the pathway involving self-efficacy and stoma self-care ability reached statistical significance, although the magnitude of this effect was small. By contrast, the indirect pathway through social support alone was not statistically significant, and the longer sequential pathway through social support, self-efficacy, and stoma self-care ability did not reach statistical significance because its 95% CI included zero. These findings suggest that self-efficacy represents the most stable mediating factor linking Type D personality traits to quality of life, while social support may contribute mainly by strengthening self-efficacy rather than by directly improving quality of life. Among the significant indirect effects, the pathway through self-efficacy alone showed the largest standardized indirect effect (-0.06), whereas pathways involving stoma self-care ability were smaller in magnitude (-0.01).

## 4 Discussion

The cross-sectional study investigated the relationships among Type D personality traits, social support, self-efficacy, stoma self-care ability, and quality of life in patients with permanent intestinal stomas. Using structural equation modeling, the study examined both the direct and indirect pathways through which personality traits may influence quality of life in this population. The findings indicate that Type D personality traits are significantly associated with lower quality of life and that this relationship is partially mediated by psychosocial and behavioral factors, including social support, self-efficacy, and stoma self-care ability.

The results of this study demonstrate that Type D personality traits are negatively associated with quality of life among patients with permanent intestinal stomas. Individuals characterized by high levels of negative affectivity and social inhibition may experience greater psychological distress and may be less likely to seek emotional or instrumental support from others. These psychological tendencies may hinder their ability to adapt effectively to the physical and social challenges associated with permanent stomas. Previous studies conducted among patients with chronic illnesses and other medical populations have also reported that Type D personality traits are associated with poorer psychological well-being and reduced health-related quality of life (21, 22). The present findings extend this body of research by demonstrating a similar association in patients living with permanent intestinal stomas. This association should also be interpreted with caution because the negative affectivity component of Type D personality traits may overlap with broader emotional distress. In patients adapting to permanent stomas, postoperative distress, body image concerns, and social embarrassment may contribute to higher DS14 scores as well as lower quality of life.

Social support was found to play an important role in the pathways linking Type D personality traits and quality of life. However, it should be noted that the hypothesized direct path from social support to quality of life did not reach statistical significance in the structural equation model (β = 0.14, *p* = 0.067). This finding suggests that social support may influence quality of life mainly through indirect psychosocial pathways rather than through a strong direct effect. Social support may provide emotional encouragement, practical assistance, and informational guidance that help patients cope with illness-related stress and adapt to the long-term lifestyle changes associated with stoma surgery (12, 23). For patients with permanent stomas, support from family members, healthcare professionals, and peer groups may be particularly important in facilitating psychological adaptation and promoting confidence in self-management (4, 7, 8, 10, 11). In the present model, social support was significantly associated with self-efficacy, and the sequential pathway involving social support and self-efficacy was statistically significant. Therefore, self-efficacy may partially or fully mediate the contribution of social support to quality of life in this population. Another possible explanation is that the direct effect of social support on quality of life may be relatively small, and the present sample size may have limited the statistical power to detect this effect, as suggested by the borderline p value. This finding does not necessarily conflict with previous studies reporting positive associations between social support and quality of life among patients with chronic diseases or ostomies (4–8, 12, 23). Rather, it suggests that when self-efficacy and self-care ability are considered simultaneously, the effect of social support may operate primarily by strengthening patients’ confidence in managing stoma-related challenges, which in turn contributes to better quality of life.

Self-efficacy was also identified as a significant mediator in the relationship between psychosocial factors and quality of life. Self-efficacy reflects individuals’ beliefs in their ability to perform behaviors necessary to manage health-related challenges (24). Patients with higher levels of self-efficacy are more likely to adopt active coping strategies and engage in effective self-management behaviors (13, 15). In the context of stoma care, individuals with greater confidence in their ability to manage stoma-related tasks may experience less anxiety and greater control over their daily lives. As a result, higher self-efficacy may contribute to improved psychological adjustment and enhanced quality of life. Recent ostomy-related studies and systematic reviews have similarly shown that interventions that improve self-efficacy or strengthen self-management behaviors may also improve adjustment and quality-of-life-related outcomes (14–18, 25). The moderate correlation between the GSES score and the OSCI self-care self-efficacy domain further suggests that generalized self-efficacy and stoma-specific self-care self-efficacy are related but distinct constructs. We retained generalized self-efficacy in the primary model because it reflects a broader psychosocial resource that may influence both perceived support and behavioral self-care capacity. In contrast, the OSCI self-care self-efficacy domain is more condition-specific and closely embedded within ostomy self-care behaviors. Therefore, the primary model used the behavioral OSCI domains to represent stoma self-care ability, while the OSCI self-care self-efficacy domain was examined in sensitivity analysis.

An important contribution of this study is the inclusion of stoma self-care ability as a behavioral factor within the mediation model. While previous studies have primarily focused on psychosocial variables such as social support and self-efficacy, relatively few investigations have examined the role of practical self-care skills in the relationship between psychological traits and quality of life. The present findings suggest that stoma self-care ability serves as an important behavioral component within the statistical pathway linking psychological resources to quality-of-life outcomes. Patients who develop stronger self-care skills are better able to manage stoma appliances, maintain peristomal skin health, and prevent complications (14, 16–18, 25). These abilities may reduce physical discomfort and increase patients’ confidence in managing their condition, thereby contributing to improved quality of life. This interpretation is supported by recent evidence demonstrating positive associations between self-care ability, telehealth-supported self-management, and quality of life among people living with a stoma (14, 16–18).

The chain mediation pathway observed in this cross-sectional model suggests complex relationships among psychological traits, social resources, behavioral capabilities, and health outcomes. Individuals with stronger Type D personality traits may be less likely to seek social interactions and emotional support, which may reduce their perceived social support. Lower levels of social support may in turn undermine patients’ confidence in their ability to manage their health condition, which may be associated with reduced self-efficacy. Reduced self-efficacy may negatively affect patients’ motivation and ability to perform effective self-care behaviors, which may be associated with poorer quality of life. Understanding this chain of relationships may help inform future psychosocial and nursing research. Recent longitudinal and qualitative studies in enterostomy populations have similarly emphasized that psychosocial adaptation, resilience, self-management needs, and sustained support are dynamically linked during recovery and long-term adjustment (10, 11, 19).

Nevertheless, because the data were cross-sectional, reverse or reciprocal relationships cannot be excluded. For example, poorer quality of life may reduce patients’ confidence in managing stoma-related tasks or negatively shape their perceptions of available support. Similarly, greater psychological distress may simultaneously increase Type D personality trait scores and reduce perceived self-efficacy. Therefore, the observed pathways should be interpreted as theoretically informed statistical associations rather than confirmed temporal processes.

The findings of this study have important clinical implications. Healthcare professionals working with patients who have permanent intestinal stomas should pay close attention to psychological characteristics such as Type D personality traits, as these traits may place patients at greater risk for poor psychosocial outcomes. Screening for psychological risk factors may help identify individuals who may benefit from additional psychosocial support. Future longitudinal and intervention studies are needed to determine whether strategies aimed at strengthening social support, self-efficacy, and stoma self-care ability can improve psychological adaptation and quality of life (12–18, 23, 25).

Several limitations of this study should be acknowledged. First, the cross-sectional design limits the ability to draw causal conclusions regarding the relationships among the study variables. Longitudinal studies are needed to further examine the temporal relationships among personality traits, psychosocial resources, self-care behaviors, and quality of life (10, 11, 19). Second, the study sample was recruited from a single tertiary hospital, which may limit the generalizability of the findings to other healthcare settings or populations. Third, the data were collected using self-reported questionnaires, which may introduce potential reporting bias. Although Harman’s single-factor test and the common latent factor analysis suggested that common method variance was unlikely to substantially bias the main findings, residual reporting bias related to self-reported measures cannot be completely excluded. Fourth, this study did not include objective clinical outcomes, such as stoma-related complications, readmissions, emergency visits, or other health indicators. Therefore, the findings primarily reflect self-reported psychosocial and quality-of-life outcomes rather than objective clinical status. Fifth, although Type D personality traits are generally conceptualized as relatively stable personality characteristics, the cross-sectional design of this study cannot distinguish stable personality traits from state-like psychological distress that may have developed or intensified after stoma surgery. Longitudinal assessments before and after surgery are needed to clarify whether lower quality of life is attributable to pre-existing Type D personality traits or to reactive psychological distress following surgery.

Despite these limitations, the present study provides valuable insights into the potential psychosocial and behavioral pathways associated with quality of life among patients with permanent intestinal stomas. By integrating personality traits, psychosocial resources, and behavioral factors into a comprehensive model, the study contributes to a deeper understanding of the complex pathways influencing patient outcomes.

## 5 Conclusion

This study showed that Type D personality traits were significantly associated with reduced quality of life among patients with permanent intestinal stomas. Social support, self-efficacy, and stoma self-care ability formed partial statistical mediation pathways in this cross-sectional SEM model. These findings highlight the importance of addressing psychological and behavioral factors in the long-term management of patients with permanent intestinal stomas. Future longitudinal and intervention studies are needed to determine whether strategies aimed at enhancing social support, strengthening self-efficacy, and improving stoma self-care ability can improve psychological adaptation and quality of life.

## Data Availability

The original contributions presented in this study are included in the article/supplementary material, further inquiries can be directed to the corresponding authors.

## References

[1] Bray F Laversanne M Sung H Ferlay J Siegel RL Soerjomataram I et al. Global cancer statistics 2022: GLOBOCAN estimates of incidence and mortality worldwide for 36 cancers in 185 countries. *CA Cancer J Clin.* (2024) 74:229–63. 10.3322/caac.21834 38572751

[2] Morgan E Arnold M Gini A Lorenzoni V Cabasag CJ Laversanne M et al. Global burden of colorectal cancer in 2020 and 2040: incidence and mortality estimates from GLOBOCAN. *Gut.* (2023) 72:338–44. 10.1136/gutjnl-2022-327736 36604116

[3] Siegel RL Wagle NS Cercek A Smith RA Jemal A. Colorectal cancer statistics, 2023. *CA Cancer J Clin.* (2023) 73:233–54. 10.3322/caac.21772 36856579

[4] Alenezi A McGrath I Kimpton A Livesay K. Quality of life among ostomy patients: a narrative literature review. *J Clin Nurs.* (2021) 30:3111–23. 10.1111/jocn.15840 33982291

[5] Ssewanyana Y Ayetey H Nyongesa C Komona R. Quality of life of adult individuals with intestinal stomas in Uganda: a cross sectional study. *Afr Health Sci.* (2021) 21:427–36. 10.4314/ahs.v21i1.53 34394325 PMC8356576

[6] Zewude WC Derese T Suga Y Teklewold B. Quality of life in patients living with stoma. *Ethiop J Health Sci.* (2021) 31:993–1000. 10.4314/ejhs.v31i5.11 35221616 PMC8843156

[7] Liu H Zhu X Yu J He P Shen B Tang X et al. The quality of life of patients with colorectal cancer and a stoma in China: a quantitative cross-sectional study. *Adv Skin Wound Care.* (2021) 34:302–7. 10.1097/01.ASW.0000744348.32773.b9 33979818

[8] Yan MH Jin Y Zhang Y Jin Y Zhang JE. Quality of life and its influencing factors among Chinese patients with permanent colostomy in the early postoperative stage: a longitudinal study. *Cancer Nurs.* (2022) 45:E153–61. 10.1097/NCC.0000000000000893 33003121

[9] Augestad KM Sneve AM Lindsetmo RO. Telemedicine in postoperative follow-up of STOMa Patients: a randomized clinical trial (the STOMPA trial). *Br J Surg.* (2020) 107:509–18. 10.1002/bjs.11491 32100297

[10] Tan Z Jiang L Lu A He X Zuo Y Yang J. Living with a permanent ostomy: a descriptive phenomenological study on postsurgical experiences in patients with colorectal cancer. *BMJ Open.* (2024) 14:e087959. 10.1136/bmjopen-2024-087959 39532360 PMC11574432

[11] Yang F Feng F Gu H Liang H Zhang J Cheng Y et al. Resilience and vulnerability of post-ostomy patients with early-onset colorectal cancer from the perspective of social-ecological theory: a qualitative study. *Front Psychiatry.* (2025) 15:1497428. 10.3389/fpsyt.2024.1497428 39906683 PMC11791536

[12] Iovino P Marcomini I Rasero L Manara DF Vellone E Villa G. Psychometric characteristics of the multidimensional scale of perceived social support in ostomy patients and their caregivers. *J Health Psychol.* (2025) 30:132–45. 10.1177/13591053241278169 39295230

[13] Chen J Tian Y Yin M Lin W Tuersun Y Li L et al. Relationship between self-efficacy and adherence to self-management and medication among patients with chronic diseases in China: a multicentre cross-sectional study. *J Psychosom Res.* (2023) 164:111105. 10.1016/j.jpsychores.2022.111105 36495756

[14] Marcomini I Iovino P Rasero L Manara DF Vellone E Villa G. Self-Care and quality of life of ostomy patients: a structural equation modeling analysis. *Nurs Rep.* (2024) 14:3417–26. 10.3390/nursrep14040247 39585138 PMC11587398

[15] Bozkul G Celik S Arslan H. Nursing interventions for the self-efficacy of ostomy patients: a systematic review. *J Tissue Viability.* (2024) 33:165–73. 10.1016/j.jtv.2024.04.006 38627154

[16] Chen HL Tu MH. The effectiveness of development mhealth apps on the self-care ability of patients with ostomy. *Stud Health Technol Inform.* (2024) 315:569–70. 10.3233/SHTI240220 39049326

[17] Qiao J Zhao Y Lu Y Li Q Dong HJ. Assessing the impact of educational eHealth and mHealth interventions on health outcomes in continuity of care for enterostomy patients: a meta-analysis. *Eur J Oncol Nurs.* (2024) 72:102676. 10.1016/j.ejon.2024.102676 39241275

[18] Bao L Jin H Lu X Wang F. Effectiveness of a community-based ostomy nursing technique to enhance the delivery of patient care and quality of life. *J Health Psychol.* (2024) 41:241–55. 10.1080/07370016.2024.2370833 38982794

[19] Li Q Lu Y Hao Y Zhao Y Qi XX Qiao J. Adaptive digital and non-digital self-management in permanent enterostomy patients: a qualitative study based on the Chronic Illness Trajectory framework. *Eur J Oncol Nurs.* (2025) 74:102733. 10.1016/j.ejon.2024.102733 39637689

[20] Denollet J. DS14: standard assessment of negative affectivity, social inhibition, and Type D personality. *Psychosom Med.* (2005) 67:89–97. 10.1097/01.psy.0000149256.81953.49 15673629

[21] Kim SR Nho JH Kim HY Ko E Jung S Kim IY et al. Type-D personality and quality of life in patients with primary brain tumours. *Eur J Cancer Care.* (2021) 30:e13371. 10.1111/ecc.13371 33184971

[22] Sánchez-Díaz M Montero-Vílchez T Quiñones-Vico MI Sierra-Sánchez Á Ubago-Rodríguez A Sanabria-de la Torre R et al. Type D personality as a marker of poorer quality of life and mood status disturbances in patients with skin diseases: a systematic review. *Acta Derm Venereol.* (2023) 103:adv00846. 10.2340/actadv.v103.2741 36625209 PMC9885290

[23] Aker FZ Karazeybek E. Relationship between perceived social support and stoma self-efficacy in permanent colostomy patients: a correlational study. *J Eval Clin Pract.* (2025) 31:e14117. 10.1111/jep.14117 39099203 PMC11656665

[24] Schwarzer R Jerusalem M. Generalized Self-Efficacy scale. In: Weinman J Wright S Johnston M editors. *Measures in Health Psychology: A User’s Portfolio. Causal and Control Beliefs.* Windsor: NFER-NELSON (1995).

[25] Wang N Zhuo H Yongni J Yan G Weihua W Jing N et al. A retrospective study examined the impact of the IKAP nursing model on the self-efficacy and quality of life of colorectal cancer patients with permanent stomas. *SAGE Open Nurs.* (2025) 11:23779608251397442. 10.1177/23779608251397442 41278385 PMC12638712

[26] Xiao SY. The theoretical basis and research application of Social Support Rating Scale. *J Clin Psychiatry.* (1994) 4:98–100.

[27] Prieto L Thorsen H Juul K. Development and validation of a quality of life questionnaire for patients with colostomy or ileostomy. *Health Qual Life Outcomes.* (2005) 3:62. 10.1186/1477-7525-3-62 16219109 PMC1274339

[28] Martín-Gil B Rivas-González N Santos-Boya T López M Jiménez JM Redondo-Pérez N et al. Changes in the quality of life of adults with an ostomy during the first year after surgery as part of the best practice spotlight organisation§programme. *Int Wound J.* (2024) 21:e14456. 10.1111/iwj.14456 37963817 PMC10898385

